# Unraveling the role of tumor sidedness in prognosis of stage II colon cancer

**DOI:** 10.1093/gastro/goae028

**Published:** 2024-04-12

**Authors:** Yun Yang, Xiaobao Yang, Zhigang Bai, Xiaozhe Gu, Saloni Rameshchandra Shah, Kenish Sirajbhai Salewala, Mansi Pankajbhai Kevadiya, Zhongtao Zhang

**Affiliations:** Department of General Surgery, Beijing Friendship Hospital, Capital Medical University, Beijing, P. R. China; State Key Lab of Digestive Health, Beijing Friendship Hospital, Beijing, P. R. China; National Clinical Research Center for Digestive Diseases, Beijing Friendship Hospital, Beijing, P. R. China; Department of General Surgery, Beijing Friendship Hospital, Capital Medical University, Beijing, P. R. China; State Key Lab of Digestive Health, Beijing Friendship Hospital, Beijing, P. R. China; National Clinical Research Center for Digestive Diseases, Beijing Friendship Hospital, Beijing, P. R. China; Department of General Surgery, Beijing Friendship Hospital, Capital Medical University, Beijing, P. R. China; State Key Lab of Digestive Health, Beijing Friendship Hospital, Beijing, P. R. China; National Clinical Research Center for Digestive Diseases, Beijing Friendship Hospital, Beijing, P. R. China; Department of General Surgery, Beijing Friendship Hospital, Capital Medical University, Beijing, P. R. China; State Key Lab of Digestive Health, Beijing Friendship Hospital, Beijing, P. R. China; National Clinical Research Center for Digestive Diseases, Beijing Friendship Hospital, Beijing, P. R. China; International School, Capital Medical University, Beijing, P. R. China; International School, Capital Medical University, Beijing, P. R. China; International School, Capital Medical University, Beijing, P. R. China; Department of General Surgery, Beijing Friendship Hospital, Capital Medical University, Beijing, P. R. China; State Key Lab of Digestive Health, Beijing Friendship Hospital, Beijing, P. R. China; National Clinical Research Center for Digestive Diseases, Beijing Friendship Hospital, Beijing, P. R. China

**Keywords:** colonic neoplasms, tumor-sidedness, right-sided colon cancer, stage II, survival

## Abstract

**Background:**

Stage II colon cancer has varying risks for metastasis, and treatment strategies depend on molecular and clinicopathological features. While tumor-sidedness is a well-accepted prognostic factor for stage III/IV colon cancer, its role in stage II is controversial. Understanding its effect in stage II is crucial for improving treatment strategies.

**Methods:**

We analyzed clinical and follow-up data of colon cancer from the Surveillance, Epidemiology, and End Results database (2004–2017). Patients were divided into a primary study cohort (2010–2017) and a validation cohort (2004–2009). The baseline characteristics between right-sided colon cancer (RCC) and left-sided colon cancer (LCC) groups were compared. Moreover, the effect of tumor size on cancer-specific survival (CSS) was evaluated using Kaplan-Meier analysis.

**Results:**

The study involved 87,355 patients in the study cohort and 65,858 in the validation cohort. Of the study cohort, 52.3% were diagnosed with RCC. The median age was 64 years old, with 48.5% females and 76.8% of white people. In addition, stage II RCC showed better CSS compared with LCC (5-year CSS 88.0% vs 85.5%, *P *<* *0.001), while stage III/IV RCC demonstrated poorer outcomes. Multivariate Cox regression analysis identified that the right-sidedness was a positive prognostic factor in stages I/II but negative in stages III (HR 1.10, *P *<* *0.001) and IV (HR 1.26, *P *<* *0.001). Chemotherapy rates decreased in RCC, particularly in stage II (RCC vs LCC: 16.2% vs 28.5%, *P *<* *0.001). Subgroup analysis, stratified by T3/T4 stages and chemotherapy status, further highlighted better survival outcomes in RCC.

**Conclusions:**

RCC is associated with a significantly better prognosis in stage II. The importance of considering tumor-sidedness in clinical decision-making and the design of future clinical trials should be emphasized.

## Introduction

With over 500,000 estimated deaths annually, colon cancer ranks as the fourth most prevalent cause of death worldwide and is also the fifth most common malignancy [1, 2]. Currently, the TNM staging system is the basis for managing patients with colon cancer. Nevertheless, this system offers limited clinical utility and moderately accurate prognostic information, particularly for stage II colon cancer [3]. Stage II colon cancer is a heterogeneous condition, comprising distinct subcategories such as low, intermediate, and high-risk groups, which are determined by prognostic factors including molecular and clinicopathological features. These factors hold immense significance in shaping treatment decisions, particularly when it comes to the use of chemotherapy. Nonetheless, the current understanding of stage II colon cancer is insufficient, necessitating further exploration of additional variables and biomarkers to improve risk classification precision and patient outcomes.

In recent studies, among the variables affecting colon cancer outcomes, tumor-sidedness has been identified as a significant prognostic factor [4]. Colon cancer can be classified into two subtypes based on anatomy and embryology: right-sided colon cancer (RCC) and left-sided colon cancer (LCC) [2], each of which has distinct clinical manifestations, genomic profiles, and survival rates. Numerous studies have consistently associated RCC with older patients, predominance of females, more advanced stages at diagnosis, and poorer differentiation [5, 6]. Furthermore, RCC and LCC differ in their genomic profiles [7], with RCC exhibiting a higher prevalence of the CpG island methylator phenotype and microsatellite instability [4, 5], while LCC displays a higher frequency of TP53 and APC gene mutations [4].

There is a widely acknowledged consensus regarding the prognostic significance of tumor-sidedness in stage IV colon cancer [8–10], and recent evidence further indicates that patients with stage III RCC typically have a poorer prognosis [11, 12]. However, the prognostic impact of tumor sidedness in early-stage colon cancer, particularly in stage II, remains controversial [13–15]. Consequently, this population-based study aims to elucidate the relationship between tumor-sidedness and cancer-specific survival (CSS) in colon cancer, particularly in stage II disease. Moreover, we seek to identify the underlying mechanisms that may explain the different prognostic implications of tumor-sidedness in early and advanced-stage colon cancer.

## Materials and methods

### Data source

With time, the Surveillance, Epidemiology, and End Results (SEER) Program has grown into an extensive network of population-based state registries that collect data concerning cancer incidence and survival from various geographic areas of the USA. We used the November 2022 submission of the SEER database for our analysis, specifically utilizing data from the SEER 17 registries.

### Patient selection

We extracted data on colon cancer patients from the SEER database using SEER*Stat software (version 8.4.2, Information Management Services, Inc, Maryland, USA). The inclusion criteria included: (i) histological confirmation of colon adenocarcinoma, mucinous adenocarcinoma, or signet-ring cell carcinoma; (ii) patients aged 18–85 years old; and (iii) diagnosis dates between 2004 and 2017. The exclusion criteria included: (i) incomplete data on survival, American Joint Committee on Cancer (AJCC) staging, or tumor site; (ii) the presence of appendiceal tumors; (iii) a survival duration of less than 30 days; and (iv) the presence of multiple primary tumors. Notably, to ensure data completeness and to account for potential confounders associated with older data, our primary study cohort comprised patients diagnosed between 2010 and 2017. In contrast, patients diagnosed between 2004 and 2009 constituted a validation cohort. This approach allowed for the validation of survival analysis results across different decades, accommodating possible variations in treatment strategies over time.

The patients were categorized as right-sided (cecum to transverse colon) or left-sided (splenic flexure to rectum), based on anatomical and embryological origins. The tumor site was determined using primary cancer site codes C18.0 to C18.9, with the splenic flexure serving as the demarcation between the right and left colon. RCC included C18.0 (cecum), C18.2 (ascending colon), C18.3 (hepatic flexure), and C18.4 (transverse colon). LCC included C18.5 (splenic flexure), C18.6 (descending colon), C18.7 (sigmoid colon), and C18.9 (rectosigmoid).

### Endpoint

The primary endpoint was cancer-specific survival (CSS). This endpoint is characterized by the proportion of patients who have died from cancer within a defined time period, while those patients who are either currently alive or have died from causes unrelated to cancer are categorized as censored.

### Statistical analyses

Quantitative data distribution was assessed using the Shapiro-Wilk test. Normally distributed data are presented as mean ± standard deviation, with the *t*-test used for intergroup comparisons. Skewed data are expressed as median (interquartile range [IQR]), analyzed using the Wilcoxon rank sum test. Frequency or percentage represented count data, compared using chi-square or Fisher's exact test as appropriate. Kaplan-Meier curves and Cox regression analysis were used to evaluate the effect of tumor site on CSS. All of the analyses were performed using the R software (version 4.3.2). A *P*-value <0.05 was considered a statistically significant difference.

## Results

### Baseline characteristics of the study cohort

Our study cohort included 87,355 patients (diagnosed between 2010 and 2017), and the validation cohort included 65,858 patients (diagnosed between 2004 and 2009). The details of baseline characteristics are described in Table 1 and Supplementary Table 1.

**Table 1.** Comparison of right-sided and left-sided colon cancer in the study cohort.

Variable	Left-sided colon cancer (*n *=* *41,691)	Right-sided colon cancer (*n *=* *45,664)	*P*-value
Female	18,708 (44.9)	23,620 (51.7)	<0.001
Age, years, median (IQR)	61 (52–70)	67 (58–75)	<0.001
Race^a^, *n* (%)			<0.001
Black	4,612 (11.1)	6,295 (13.8)	
White	31,325 (75.6)	35,407 (77.9)	
Other	5,500 (13.3)	3,767 (8.3)	
Histology, *n* (%)			<0.001
Adenocarcinoma	39,266 (94.2)	40,292 (88.2)	
Mucinous adenocarcinoma	2,148 (5.2)	4,744 (10.4)	
Signet-ring cell adenocarcinoma	277 (0.7)	628 (1.4)	
Chemotherapy, *n* (%)			<0.001
No/unknown	21,825 (52.3)	28,012 (61.3)	
Yes	19,866 (47.7)	17,652 (38.7)	
Size, cm, median (IQR)	4.10 (2.90–5.80)	4.50 (3.00–6.40)	<0.001
Grade^b^, *n* (%)			<0.001
High/moderate	34,628 (85.7)	34,202 (76.8)	
Poor/anaplastic	5,764 (14.3)	10,356 (23.2)	
AJCC stage, *n* (%)			<0.001
I	8,816 (21.1)	9,668 (21.2)	
II	11,130 (26.7)	14,545 (31.9)	
III	14,549 (34.9)	14,305 (31.3)	
IV	7,196 (17.3)	7,146 (15.6)	

AJCC = American Joint Committee on Cancer, IQR = interquartile range.

a Due to the missing data, the total is 86,906.

b Due to the missing data, the total is 84,950.

In the study cohort, participants were monitored for a median duration of 79 months, exhibiting an IQR of 56 to 105 months. During this period, 27.5% of the patients (24,033 out of 87,355) reached the primary endpoint. The median age of the patients was 64 years old (IQR 55–73), with 48.5% of them being female, and 76.8% being white people. Patients with RCC constituted 52.3% of the total cohort. Based on detailed tumor location frequencies, the sigmoid colon had the highest occurrence (27.7%), followed by the cecum (20.8%) and the ascending colon (18.7%). Histopathological classification showed that the majority of the cases were of adenocarcinoma (91.1%), with mucinous cell adenocarcinoma (7.9%) and signet-ring cell carcinoma (1.0%) following in order of prevalence. Approximately 19.0% of all tumors exhibited poor or undifferentiated histology (Grade 3–4), and 16.4% of all patients presented with metastatic disease at diagnosis.

### Comparative analysis of RCC vs LCC

A comprehensive comparison between RCC and LCC is presented in Table 1 and Supplementary Table 2. Patients with RCC were observed to be significantly older (median age, 67 vs 61 years old, *P *<* *0.001), with a higher proportion of females (51.7% vs 44.9%, *P *<* *0.001). These patients exhibited a greater frequency of poorly differentiated or anaplastic tumors (23.2% vs 14.3%, *P *<* *0.001) and a higher incidence of mucinous adenocarcinoma or signet-ring cell carcinoma (11.8% vs 5.8%, *P *<* *0.001). Furthermore, tumors in RCC patients were significantly larger (median size, 4.50 vs 4.10 cm, *P *<* *0.001).

### Tumor sidedness and its impact on cancer-specific survival

**Figure 1.**
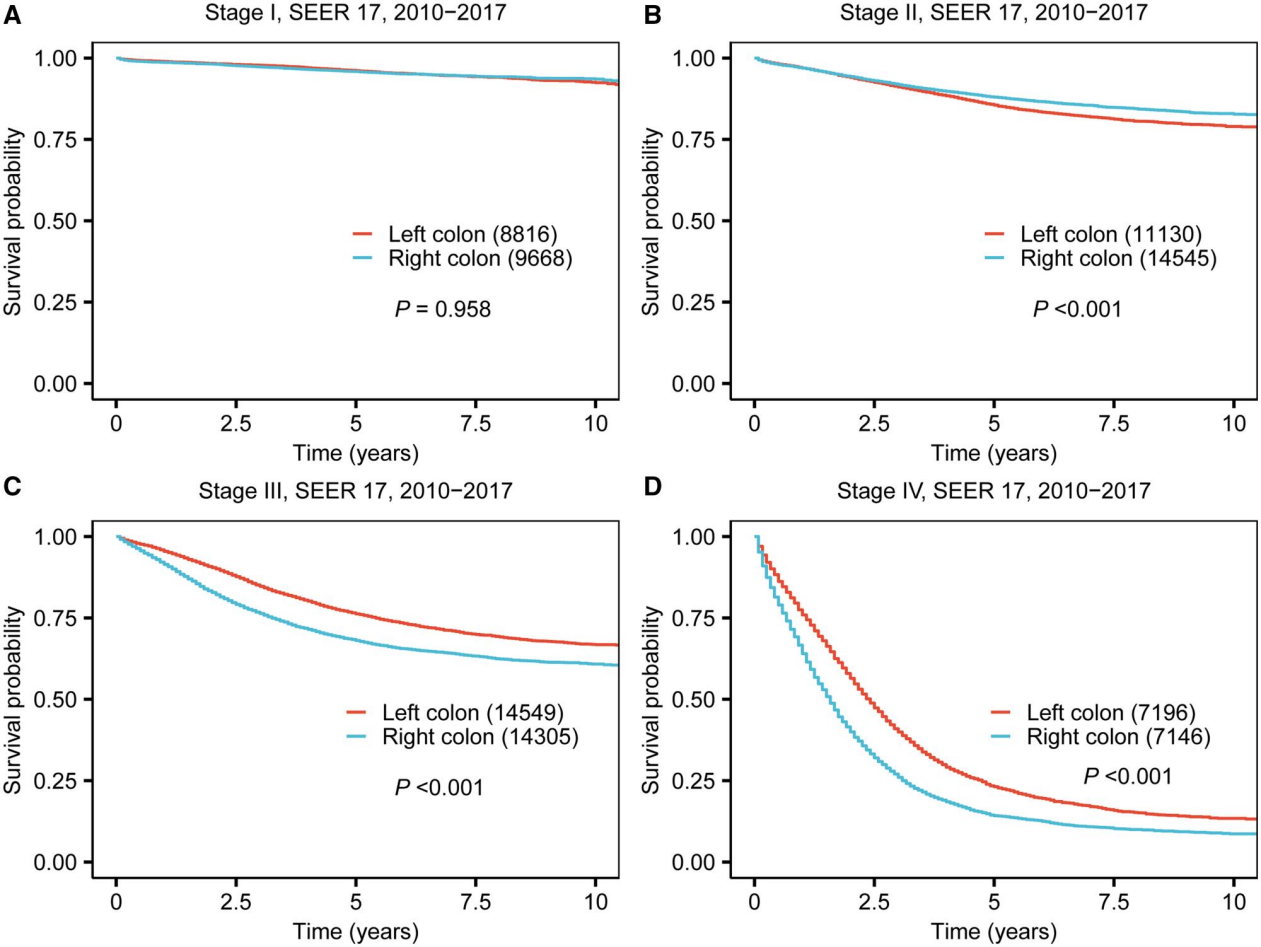
Kaplan-Meier curve of prognosis difference between right-sided and left-sided colon cancer across four stages for patients diagnosed between 2010 and 2017. (A) Stage I. (B) Stage II. (C) Stage III. (D) Stage IV.

**Figure 2.**
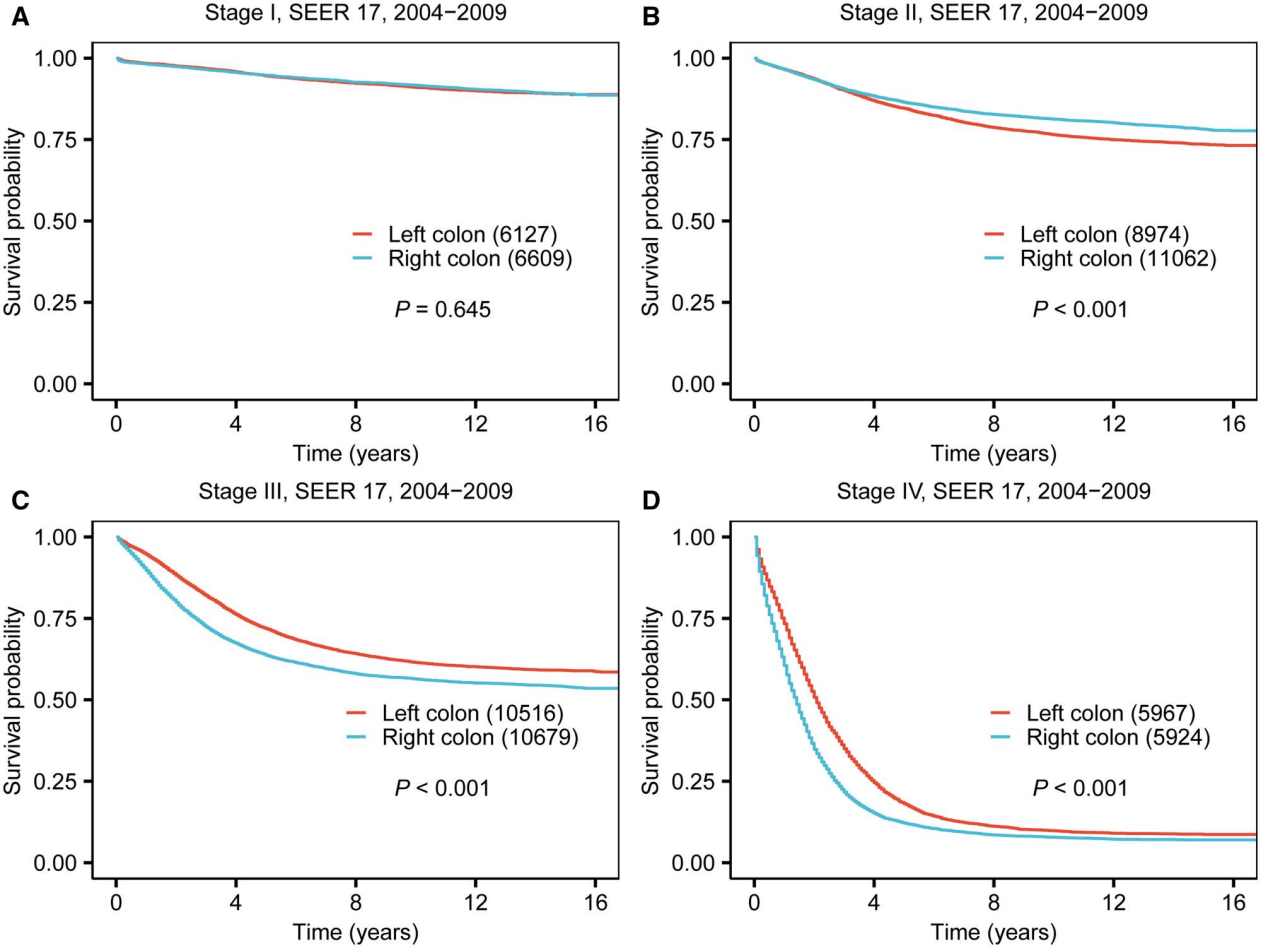
Kaplan-Meier curve of prognosis difference between right-sided and left-sided colon cancer across four stages for patients diagnosed between 2004 and 2009. (A) Stage I. (B) Stage II. (C) Stage III. (D) Stage IV.

The survival analysis of the overall study cohort demonstrated significantly poorer 5-year CSS rates for RCC compared with LCC (68.2% vs 69.7%, *P *<* *0.001). This finding was similar in the validation cohort, as depicted in Supplementary Figure 1. Stratifying the data by tumor location and AJCC tumor stage, our study uncovered notable disparities in CSS, which are illustrated in Kaplan-Meier survival curves (Figure 1 and Supplementary Figure 2), and quantified in Table 2 with 5-year and 10-year CSS rates. In stage I, the difference in CSS between RCC and LCC was not statistically significant (5-year CSS 95.9% for RCC vs 96.1% for LCC, *P *=* *0.958). However, in stage II, RCC patients exhibited a notably better prognosis compared with those with LCC (5-year CSS rate 88.0% vs 85.5%, *P *<* *0.001). In contrast, stages III and IV RCC patients showed a significantly worse prognosis compared to those with LCC (5-year CSS 68.1% vs 76.2%, *P *<* *0.001 for stage III; 14.2% vs 23.1%, *P *<* *0.001 for stage IV). Further analysis performed with the validation cohort confirmed these trends for RCC (Figure 2), indicating a more favorable prognosis in stage II, but an adverse prognosis in more advanced stages (stage III/IV).

**Table 2.** Five-year and ten-year CSS rates for the patients stratified by AJCC stage.

Variable	Stage I			Stage II			Stage III			Stage IV		
	5-year CSS (95% CI)	10-year CSS (95% CI)	*P*-value	5-year CSS (95% CI)	10-year CSS (95% CI)	*P*-value	5-year CSS (95% CI)	10-year CSS (95% CI)	*P*-value	5-year CSS (95% CI)	10-year CSS (95% CI)	*P*-value
Sidedness			0.958			<0.001			<0.001			<0.001
Left colon	96.1 (95.7, 96.6)	92.4 (91.6, 93.3)		85.5 (84.8, 86.2)	79.0 (77.9, 80.0)		76.2 (75.5, 77.0)	66.8 (65.7, 67.8)		23.1 (22.1, 24.2)	13.4 (12.3, 14.6)	
Right colon	95.9 (95.4, 96.3)	93.5 (92.8, 94.2)		88.0 (87.4, 88.6)	82.8 (81.9, 83.6)		68.1 (67.2, 68.9)	60.8 (59.8, 61.9)		14.2 (13.4, 15.2)	8.64 (7.71, 9.68)	

CSS = cancer-specific survival, AJCC = American Joint Committee on Cancer, CI = confidence interval.

Multivariate Cox regression analysis reinforced tumor-sidedness as an independent prognostic factor in colon cancer. Specifically, in stages I and II, RCC emerged as a favorable prognostic factor (HR 0.75, *P *<* *0.001 for stage I; HR 0.69, *P *<* *0.001 for stage II). Conversely, in stages III and IV, RCC was identified as an unfavorable prognostic factor (HR 1.10, *P *<* *0.001 for stage III; HR 1.26, *P *<* *0.001 for stage IV) (Table 3).

**Table 3.** Multivariate cox analysis for the patients stratified by AJCC stage.

Variable	Stage I			Stage II			Stage III			Stage IV		
	HR	95% CI	*P*-value	HR	95% CI	*P*-value	HR	95% CI	*P*-value	HR	95% CI	*P*-value
Age, 1 year increased	1.05	1.04, 1.06	<0.001	1.03	1.03, 1.04	<0.001	1.02	1.02, 1.02	<0.001	1.02	1.01, 1.02	<0.001
Gender (male vs female)	1.23	1.07, 1.41	0.004	1.10	1.03, 1.18	0.005	1.11	1.06, 1.16	<0.001	1.04	1.00, 1.08	0.046
Sidedness (right vs left)	0.75	0.65, 0.87	<0.001	0.69	0.65, 0.74	<0.001	1.10	1.05, 1.15	<0.001	1.26	1.21, 1.31	<0.001
Size, 1 cm increased	1.15	1.12, 1.19	<0.001	1.07	1.06, 1.08	<0.001	1.07	1.06, 1.08	<0.001	1.03	1.02, 1.04	<0.001
Grade (poor/anaplastic vs high/moderate)	1.10	0.86, 1.42	0.439	1.08	0.99, 1.19	0.095	1.51	1.44, 1.59	<0.001	1.42	1.36, 1.48	<0.001
Histology												
Adenocarcinoma	1	Reference	–	1	Reference	–	1	Reference	–	1	Reference	–
Mucinous adenocarcinoma	0.99	0.71, 1.37	0.951	1.07	0.96, 1.20	0.234	1.21	1.12, 1.30	<0.001	1.09	1.02, 1.17	0.012
Signet-ring cell adenocarcinoma	0.51	0.07, 3.66	0.503	1.35	0.90, 2.03	0.150	2.18	1.92, 2.48	<0.001	1.50	1.31, 1.71	<0.001

AJCC = American Joint Committee on Cancer, CI = confidence interval, HR = hazard ratio.

### Stage-specific clinical feature comparison of RCC and LCC

To elucidate the mechanisms underlying the differential impact of tumor sidedness on stages II and III/IV colon cancer, we conducted an extensive comparative analysis of the clinical characteristics between RCC and LCC throughout all four stages (Table 4).

**Table 4.** Comparison of clinical features of right-sided and left-sided colon cancer

Variable	Stage I			Stage II			Stage III			Stage IV		
	Left-sided colon cancer (*n *=* *8,816）	Right-sided colon cancer (*n *=* *9,668)	*P*-value	Left-sided colon cancer (*n *=* *11,130)	Right-sided colon cancer (*n *=* *14,545)	*P*-value	Left-sided colon cancer (*n *=* *14,549)	Right-sided colon cancer (*n *=* *14,305)	*P*-value	Left-sided colon cancer (*n *=* *7,196)	Right-sided colon cancer (*n *=* *7,146)	*P*-value
Female, *n* (%)	3,994 (45.3)	5,024 (52.0)	<0.001	4,932 (44.3)	7,457 (51.3)	<0.001	6,572 (45.2)	7,526 (52.6)	<0.001	3,210 (44.6)	3,613 (50.6)	<0.001
Age, years, median (IQR)	62 (53–70)	69 (60–76)	<0.001	63 (54–72)	68 (59–77)	<0.001	60 (51–69)	66 (57–75)	<0.001	59 (50–68)	64 (55–73)	<0.001
Histology, *n* (%)			<0.001			<0.001			<0.001			<0.001
Adenocarcinoma	8,610 (97.7)	9,087 (94.0)		10,414 (93.6)	12,659 (87.0)		13,601 (93.5)	12,408 (86.7)		6,641 (92.3)	6,138 (85.9)	
Mucinous adenocarcinoma	194 (2.2)	556 (5.8)		688 (6.1)	1,789 (12.3)		809 (5.6)	1,587 (11.1)		457 (6.4)	812 (11.4)	
Signet-ring cell adenocarcinoma	12 (0.1)	25 (0.3)		28 (0.3)	97 (0.7)		139 (1.0)	310 (2.2)		98 (1.4)	196 (2.7)	
Chemotherapy, *n* (%)			<0.001			<0.001			<0.001			<0.001
No/unknown	8,648 (98.1)	9,595 (99.2)		7,956 (71.5)	12,186 (83.8)		3,674 (25.3)	4,317 (30.2)		1,547 (21.5)	1,914 (26.8)	
Yes	168 (1.9)	73 (0.8)		3,174 (28.5)	2,359 (16.2)		10,875 (74.7)	9,988 (69.8)		5,649 (78.5)	5,232 (73.2)	
Size, median (IQR)	2.00 (1.00–3.50)	2.50 (1.40–4.00)	<0.001	5.00 (3.50–6.50)	5.00 (3.60–7.00)	<0.001	4.30 (3.00–5.70)	4.90 (3.50–6.50)	<0.001	5.00 (4.00–6.50)	5.30 (4.00–7.00)	<0.001
Grade^a^, *n* (%)			<0.001			<0.001			<0.001			<0.001
High/moderate	7,885 (93.6)	8,598 (91.4)		9,847 (90.1)	11,679 (81.5)		11,776 (82.2)	9,645 (68.4)		5,120 (76.3)	4,280 (63.7)	
Poor/anaplastic	535 (6.4)	814 (8.6)		1,088 (9.9)	2,648 (18.5)		2,555 (17.8)	4,458 (31.6)		1,586 (23.7)	2,436 (36.3)	
T stage, *n* (%)			<0.001			<0.001			<0.001			<0.001
T1	4,749 (53.9)	4,315 (44.6)					674 (4.6)	424 (3.0)		404 (5.6)	326 (4.6)	
T2	4,067 (46.1)	5,353 (55.4)					1,413 (9.7)	1,141 (8.0)		176 (2.4)	161 (2.3)	
T3				9,068 (81.5)	12,362 (85.0)		9,405 (64.6)	9,105 (63.6)		3,634 (50.5)	3,317 (46.4)	
T4				2,062 (18.5)	2,183 (15.0)		3,057 (21.0)	3,635 (25.4)		2,982 (41.4)	3,342 (46.8)	
N stage, *n* (%)									<0.001			<0.001
N0	8,816 (100.0)	9,668 (100.0)		11,130 (100.0)	14,545 (100.0)					1,625 (22.6)	1,142 (16.0)	
N1							9,780 (67.2)	9,246 (64.6)		2,836 (39.4)	2,458 (34.4)	
N2							4,769 (32.8)	5,059 (35.4)		2,735 (38.0)	3,546 (49.6)	

IQR = interquartile range.

a Due to the missing data, the total is 84,950.

Consistent with observations in stages III/IV, those with stage II RCC were generally older (median ages were 68 vs 63 years old, *P *<* *0.001), had a higher occurrence of mucinous adenocarcinoma or signet-ring cell carcinoma (13.0% vs 6.4%, *P *<* *0.001), and a higher prevalence of poorly differentiated or anaplastic carcinoma (18.5% vs 9.9%, *P *<* *0.001). These factors, traditionally associated with a poorer prognosis, run counter to the observed favorable prognosis in stage II RCC.

Interestingly, a notable finding was that there were consistently lower rates of chemotherapy utilization in RCC compared with LCC across all evaluated stages. Specifically, for stage II, the rates were 16.2% in RCC vs 28.5% in LCC (*P *<* *0.001); for stage III, 69.8% vs 74.7% (*P *<* *0.001); and for stage IV, 73.2% vs 78.5% (*P *<* *0.001). This difference was most pronounced in stage II, with a disparity of 12.3% between RCC and LCC, followed by stage III (4.9%) and stage IV (5.3%). Additionally, in stage II, the data indicated a lower incidence of T4 tumors in RCC compared with LCC (15.0% vs 18.5%, *P *<* *0.001).

### Subgroup analysis of survival impact based on sidedness in stage II Colon cancer

**Figure 3.**
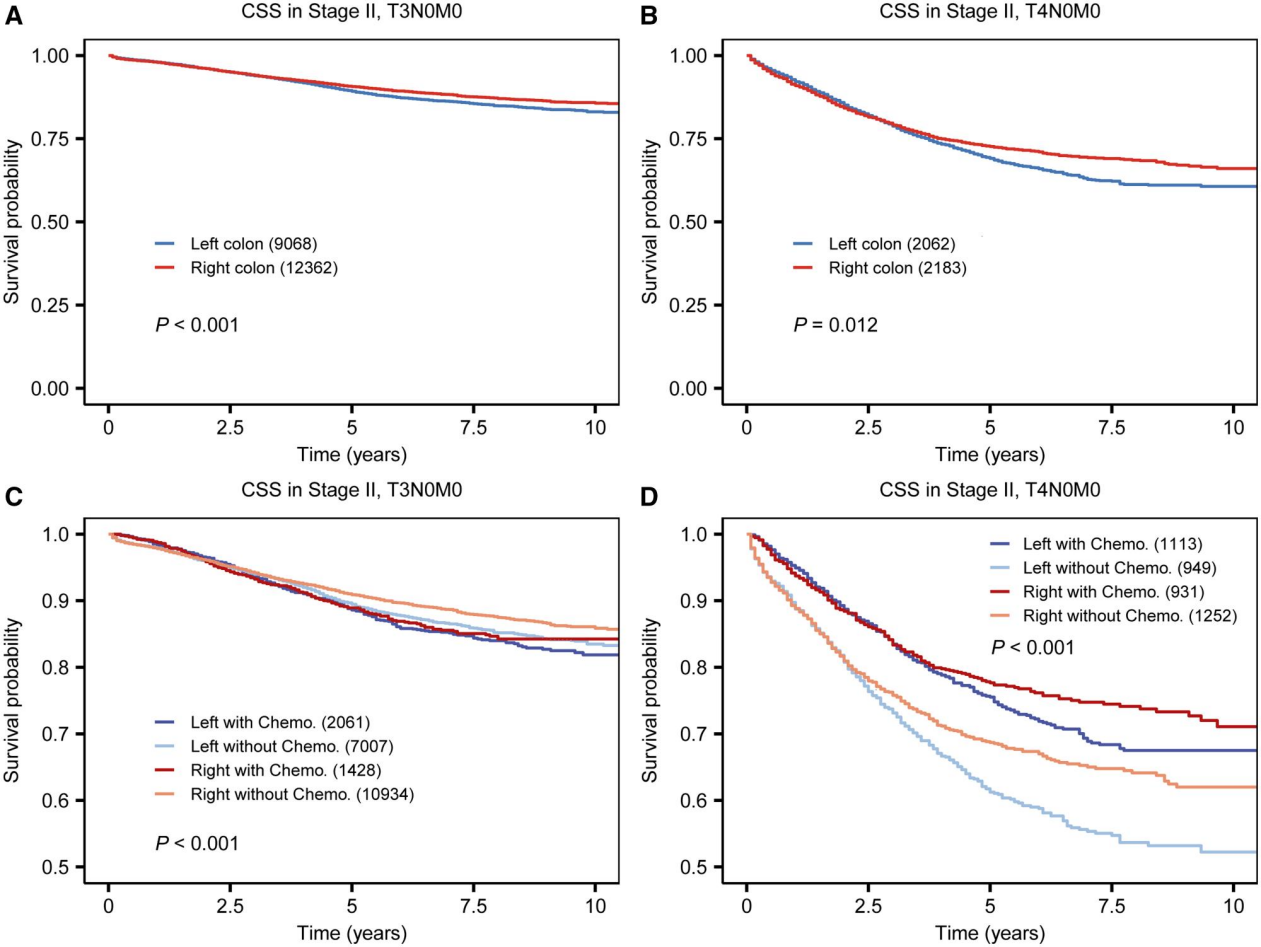
Subgroup survival analysis of stage II patients stratified by T stage and chemotherapy status. Kaplan-Meier survival curve comparing the prognosis of RCC and LCC in (A) T3N0M0, (B) T4N0M0 patients, Kaplan-Meier curve of prognosis of RCC and LCC stratified by chemotherapy status, in (C) T3N0M0, and (D) T4N0M0 patients. CSS = cancer-specific survival, RCC = right-sided colon cancer, LCC = left-sided colon cancer, Chemo. = chemotherapy.

Given that both chemotherapy and T4 stage are significant prognostic factors for stage II colon cancer patients, we conducted a subgroup analysis stratified by T stages and chemotherapy status (Figure 3). The Kaplan-Meier plots reveal a consistently better survival rate for RCC compared with LCC. Specifically, for T3N0M0 patients, the 5-year CSS was 90.7% for RCC vs 89.3% for LCC (*P *<* *0.001; Figure 3A and B). Further, stratification by both T stages and chemotherapy status (Figure 3C and D) indicates that RCC patients without chemotherapy at the T3 stage exhibit the highest survival rate, with a 5-year CSS of 90.9% (95% CI: 90.3%–91.5%). In T4N0M0 patients, those with RCC receiving chemotherapy showed the best survival outcomes (5-year CSS of 77.7% for RCC with chemotherapy, compared with 75.5% for LCC with chemotherapy, 68.7% for RCC without chemotherapy, and 61.2% for LCC without chemotherapy, all *P *<* *0.001).

## Discussion

Colon cancer is one of the leading causes of cancer-related deaths worldwide [2]. Currently, histopathological assessments play a major role in patient management and prediction of clinical outcomes. Nevertheless, stage II colon cancer comprises a heterogeneous group of low-, intermediate- and high-risk diseases for metastatic dissemination in contrast to stage III and IV disease [3]. Risk stratification is based on assessments of high-risk factors including various factors, including poorly differentiated or undifferentiated histology, lymphovascular invasion, perineural invasion, as well as low-risk factors such as microsatellite instability-high (MSI-H) status [16]. These factors are closely associated with treatment decision-making, such as the application of adjuvant chemotherapy, which is typically recommended for patients with high risks. However, the relevance of tumor-sidedness in stage II colon cancer remains controversial. Elucidating the potential prognostic utility of sidedness could augment risk stratification and guide clinical decision-making and trial design for this heterogeneous patient population.

In this study, we evaluated the impact of tumor sidedness on the survival of patients across four stages of colon cancer, using a substantial cohort from the SEER database. Employing Kaplan-Meier methods and multivariate Cox regression models, we found that RCC correlates with a significantly better prognosis in stage II. However, this trend reverses in stages III and IV, where RCC is associated with poorer clinical outcomes. Recent research has increasingly focused on the prognostic implications of primary tumor sidedness in early-stage colorectal cancer. Ouchi *et al*. [15] analyzed a cohort of 4,113 patients from four randomized controlled trials (JCOG2003A) with pathological stage II–III adenocarcinoma of the colon. This analysis, which compared relapse-free survival post-primary surgery in stage II patients, found 5-year relapse-free survival rates for RCC and LCC to be 89.7% and 86.9% respectively. However, these differences did not reach statistical significance (*P *=* *0.2385). The limited sample size of 809 stage II patients and a maximum follow-up period of five years in this cohort might have contributed to the lack of statistical significance. In contrast, a meta-analysis [14] encompassing 581,542 stage I–III colon cancer patients from 37 studies reported that right-sided tumors were associated with improved overall survival (HR, 0.89; 95% CI 0.86–0.92) and CSS (HR 0.78; 95% CI 0.70–0.86) in stage II cancer patients. This finding is consistent with our observations. Furthermore, another study [17] involving 114,839 patients diagnosed with stage I and II colon cancer from the National Cancer Database in America indicated that left-sided tumors were linked to poorer survival (HR 1.13; 95% CI 1.09–1.17, *P *<* *0.001). Both of these studies have an extensive dataset and support our findings.

To elucidate the mechanisms underlying the better survival observed in stage II colon cancer, we conducted a comprehensive analysis across all stages. This analysis uncovered a consistently lower chemotherapy administration rate in patients at all stages, particularly pronounced in those with stage II disease. According to current guidelines [18, 19], all the patients of stage III/IV are recommended for systemic treatment, while adjuvant chemotherapy is not routinely advised for stage II colon cancer, with MSI-H status being a pivotal factor in chemotherapy decision-making [20]. For stage III/IV patients, the advanced age of RCC and anticipated treatment intolerance may contribute to their marginally reduced chemotherapy rates [21]. In stage II, the variance in MSI-H status, influenced by tumor sidedness and disease stage, appears to be a key factor. The PETACC-3 trial data show a higher prevalence of MSI-H tumors in stage II compared with stage III (22% vs 12%, *P *<* *0.001) [22], while in stage IV, the prevalence of MSI-H (dMMR) tumors is even lower, ranging from 3.5% to 6.5% in various studies [23–25]. Additionally, a significant disparity exists between RCC and LCC in MSI-H rates, with stage II and stage III diseases showing higher prevalence in RCC (stage II: right vs left 29.9% vs 13.1%; stage III: right vs left 24.6% vs 10.1%) [26]. These observations might account for the lower chemotherapy rates in stage II RCC and elucidate the differential survival impacts of tumor sidedness across various stages. These studies suggest that a greater percentage of MSI-H patients, a low-risk factor, appear to be in stage II RCC. Better RCC survival may also be attributed to MSI-H patients' decreased treatment requirement and avoidance of chemotherapy toxicity [27].

As T4 is currently recognized as a major prognostic factor associated with worse survival [28], we performed a subgroup analysis, stratified by T stages and chemotherapy status, which revealed that RCC is associated with improved survival in both T3 and T4 stage II colon cancer patients. Interestingly, the survival advantage persists even after further categorizing T4 patients based on their chemotherapy status, underscoring the prognostic significance of RCC. Furthermore, the evidence indicating improved survival for T4 patients who underwent chemotherapy, regardless of tumor sidedness, suggests that chemotherapy should be considered a viable treatment option for T4 stage colon cancer patients, which is consistent with the latest guideline [18].

In addition to our findings regarding the prognostic impact of tumor sidedness in stage II colon cancer, further advancements must be made in the identification of tumors and biological biomarkers. Notably, mutations in BRAF have emerged as potential risk factors [29], while gene-expression panel tests like ColoPrint and ColDX offer promising tools for personalized risk assessment [30, 31]. Moreover, circulating tumor DNA has garnered significant interest as a potentially transformative tool for risk stratification in stage II disease [32].

There were certain limitations to our study. First, the retrospective nature of the analysis, relying on the SEER database, may have inherent biases due to the observational study design. Second, the lack of detailed treatment data, including chemotherapy regimens and surgical procedures, limits our ability to fully understand the impact of these factors on survival outcomes. Third, there was inadequate information in the SEER database regarding the potential for molecular and genetic differences between right-sided and left-sided colon cancers, which could influence prognosis and response to therapy. As a result, these differences could not be evaluated directly. All these limitations underscore the need for prospective studies to validate our findings and delve deeper into the underlying biological mechanisms further.

## Conclusions

This study provides compelling evidence of the independent influence of primary tumor sidedness on stage II colon cancer, with significantly better survival in RCC. The survival disparities and underlying mechanisms highlight the importance of considering tumor-sidedness in clinical decision-making and the design of future clinical trials.

## Supplementary Material

goae028_Supplementary_Data
