# Supplementary material for: Unraveling the role of tumor sidedness in prognosis of stage II colon cancer
Source: Gastroenterol Rep (Oxf). 2024 Apr 12;12:goae028. doi: 10.1093/gastro/goae028 (PMC11014780; doi:10.1093/gastro/goae028)
Supplement: goae028_Supplementary_Data [file goae028_supplementary_data.zip › Supplementary Material final version 20240320.docx]

Supplementary Material

Supplementary table 1. Baseline clinicopathological characteristics of included patients in SEER 17.

| Characteristic | 2004–2009 (*n* = 65,858) | 2010–2017 (*n* = 87,355) | *P*-value |
| --- | --- | --- | --- |
| Gender, *n* (%) |  |  | <0.001 |
| Female | 32,822 (49.8) | 42,328 (48.5) |  |
| Male | 33,036 (50.2) | 45,027 (51.5) |  |
| Age, years, median (IQR) | 66 (56–75) | 64 (55–73) | <0.001 |
| Race^a^, *n* (%) |  |  | <0.001 |
| Black | 7,727 (11.8) | 10,907 (12.6) |  |
| White | 51,952 (79.0) | 66,732 (76.8) |  |
| Other | 6,051 (9.2) | 9,267 (10.7) |  |
| Marital status, *n* (%) |  |  | <0.001 |
| Married | 39,488 (60.0) | 50,477 (57.8) |  |
| Not married | 26,370 (40.0) | 36,878 (42.2) |  |
| Histology, *n* (%) |  |  | <0.001 |
| Adenocarcinoma | 58,348 (88.6) | 79,558 (91.1) |  |
| Mucinous adenocarcinoma | 6,802 (10.3) | 6,892 (7.9) |  |
| Signet-ring cell adenocarcinoma | 708 (1.1) | 905 (1.0) |  |
| CEA^b^, *n* (%) |  |  |  |
| Negative | NA | 30,903 (56.2) |  |
| Positive | NA | 24,050 (43.8) |  |
| Location, *n* (%) |  |  | <0.001 |
| Ascending colon | 11,442 (17.4) | 16,292 (18.7) |  |
| Cecum | 14,342 (21.8) | 18,205 (20.8) |  |
| Descending colon | 3,721 (5.7) | 5,239 (6.0) |  |
| Hepatic flexure | 3,087 (4.7) | 3,573 (4.1) |  |
| Rectosigmoid junction | 7,434 (11.3) | 9,492 (10.9) |  |
| Sigmoid colon | 18,131 (27.5) | 24,205 (27.7) |  |
| Splenic flexure | 2,298 (3.5) | 2,755 (3.2) |  |
| Transverse colon | 5,403 (8.2) | 7,594 (8.7) |  |
| Location (grouped), *n* (%) |  |  | 0.369 |
| Left-sided colon cancer | 31,584 (48.0) | 41,691 (47.7) |  |
| Right-sided colon cancer | 34,274 (52.0) | 45,664 (52.3) |  |
| Surgery^c^, *n* (%) |  |  | <0.001 |
| No surgery | 835 (1.3) | 2,192 (2.5) |  |
| Surgery | 65,010 (98.7) | 85,147 (97.5) |  |
| Chemotherapy, *n* (%) |  |  | <0.001 |
| No/unknown | 39,061 (59.3) | 49,837 (57.1) |  |
| Yes | 26,797 (40.7) | 37,518 (42.9) |  |
| Size, cm, median (IQR) | 4.30 (3.00–6.00) | 4.50 (3.00–6.00) | 0.171 |
| Grade^d^, *n* (%) |  |  | <0.001 |
| High/moderate | 50,700 (78.9) | 68,830 (81.0) |  |
| Poor/anaplastic | 13,550 (21.1) | 16,120 (19.0) |  |
| PNI^e^, *n* (%) |  |  |  |
| Negative | NA | 68,789 (86.4) |  |
| Positive | NA | 10,854 (13.6) |  |
| Tumor deposit, *n* (%) |  |  |  |
| Negative | NA | 68,838 (84.9) |  |
| Positive | NA | 12,215 (15.1) |  |
| T stage, *n* (%) |  |  | <0.001 |
| T1 | 6,279 (9.5) | 10,892 (12.5) |  |
| T2 | 9,653 (14.7) | 12,311 (14.1) |  |
| T3 | 39,700 (60.3) | 46,891 (53.7) |  |
| T4 | 10,226 (15.5) | 17,261 (19.8) |  |
| N stage, *n* (%) |  |  | <0.001 |
| N0 | 34,924 (53.0) | 46,926 (53.7) |  |
| N1 | 17,367 (26.4) | 24,320 (27.8) |  |
| N2 | 13,567 (20.6) | 16,109 (18.4) |  |
| M stage, *n* (%) |  |  | <0.001 |
| M0 | 53,967 (81.9) | 73,013 (83.6) |  |
| M1 | 11,891 (18.1) | 14,342 (16.4) |  |
| AJCC stage, *n* (%) |  |  | <0.001 |
| Ⅰ | 12,736 (19.3) | 18,484 (21.2) |  |
| Ⅱ | 20,036 (30.4) | 25,675 (29.4) |  |
| Ⅲ | 21,195 (32.2) | 28,854 (33.0) |  |
| Ⅳ | 11,891 (18.1) | 14,342 (16.4) |  |

IQR, interquartile range; NA, not available; CEA, serum carcinoembryonic antigen level; PNI, perineural invasion; AJCC, American Joint Committee on Cancer.

^a^Due to the missing data, the total is 152,636.

^b^Due to the missing data, the total is 54,953.

^c^Due to the missing data, the total is 153,184.

^d^Due to the missing data, the total is 149,200.

^e^Due to the missing data, the total is 79,643.

Supplementary table 2. Comparison of right-sided and left-sided colon cancer in cohort 2010–2017

(*n* = 87,355; complete version of Table 1)

| Variable | Left-sided colon cancer  (*n* = 41,691) | Right-sided colon cancer  (*n* = 45,664) | *P*-value |
| --- | --- | --- | --- |
| Gender, *n* (%) |  |  | <0.001 |
| Female | 18,708 (44.9) | 23,620 (51.7) |  |
| Male | 22,983 (55.1) | 22,044 (48.3) |  |
| Age, years, median (IQR) | 61 (52–70) | 67 (58–75) | <0.001 |
| Race^a^, *n* (%) |  |  | <0.001 |
| Black | 4,612 (11.1) | 6,295 (13.8) |  |
| White | 31,325 (75.6) | 35,407 (77.9) |  |
| Other | 5,500 (13.3) | 3,767 (8.3) |  |
| Income^b^, *n* (%) |  |  | <0.001 |
| Low | 1,648 (4.0) | 1,868 (4.1) |  |
| Median | 25,727 (61.7) | 29,122 (63.8) |  |
| High | 14,315 (34.3) | 14,673 (32.1) |  |
| Marital status, *n* (%) |  |  | <0.001 |
| Married | 24,447 (58.6) | 26,030 (57.0) |  |
| Not married | 17,244 (41.4) | 19,634 (43.0) |  |
| Histology, *n* (%) |  |  | <0.001 |
| Adenocarcinoma | 39,266 (94.2) | 40,292 (88.2) |  |
| Mucinous adenocarcinoma | 2,148 (5.2) | 4,744 (10.4) |  |
| Signet-ring cell | 277 (0.7) | 628 (1.4) |  |
| CEA^c^, *n* (%) |  |  | <0.001 |
| Negative | 14,440 (55.0) | 16,463 (57.4) |  |
| Positive | 11,836 (45.0) | 12,214 (42.6) |  |
| Surgery^d^, *n* (%) |  |  | <0.001 |
| No surgery | 1,299 (3.1) | 893 (2.0) |  |
| Surgery | 40,381 (96.9) | 44,766 (98.0) |  |
| Chemotherapy, *n* (%) |  |  | <0.001 |
| No/unknown | 21,825 (52.3) | 28,012 (61.3) |  |
| Yes | 19,866 (47.7) | 17,652 (38.7) |  |
| Size, cm, median (IQR) | 4.10 (2.90–5.80) | 4.50 (3.00–6.40) | <0.001 |
| Grade^e^, *n* (%) |  |  | <0.001 |
| High/moderate | 34,628 (85.7) | 34,202 (76.8) |  |
| Poor/anaplastic | 5,764 (14.3) | 10,356 (23.2) |  |
| PNI^f^, *n* (%) |  |  | <0.001 |
| Negative | 31,932 (84.9) | 36,857 (87.7) |  |
| Positive | 5,673 (15.1) | 5,181 (12.3) |  |
| Tumor deposit^g^, *n* (%) |  |  | <0.001 |
| Negative | 31,924 (83.8) | 36,914 (86.0) |  |
| Positive | 6,192 (16.2) | 6,023 (14.0) |  |
| T stage, *n* (%) |  |  | <0.001 |
| T1 | 5,827 (14.0) | 5,065 (11.1) |  |
| T2 | 5,656 (13.6) | 6,655 (14.6) |  |
| T3 | 22,107 (53.0) | 24,784 (54.3) |  |
| T4 | 8,101 (19.4) | 9,160 (20.1) |  |
| N stage, *n* (%) |  |  | <0.001 |
| N0 | 21,571 (51.7) | 25,355 (55.5) |  |
| N1 | 12,616 (30.3) | 11,704 (25.6) |  |
| N2 | 7,504 (18.0) | 8,605 (18.8) |  |
| M stage, *n* (%) |  |  | <0.001 |
| M0 | 34,495 (82.7) | 38,518 (84.4) |  |
| M1 | 7,196 (17.3) | 7,146 (15.6) |  |
| AJCC stage, *n* (%) |  |  | <0.001 |
| Ⅰ | 8,816 (21.1) | 9,668 (21.2) |  |
| Ⅱ | 11,130 (26.7) | 14,545 (31.9) |  |
| Ⅲ | 14,549 (34.9) | 14,305 (31.3) |  |
| Ⅳ | 7,196 (17.3) | 7,146 (15.6) |  |

IQR, interquartile range; CEA, serum carcinoembryonic antigen level; PNI, perineural invasion; AJCC, American Joint Committee on Cancer.

^a^Due to the missing data, the total is 86,906.

^b^Due to the missing data, the total is 87,353.

^c^Due to the missing data, the total is 54,953.

^d^Due to the missing data, the total is 87,339.

^e^Due to the missing data, the total is 84,950.

^f^Due to the missing data, the total is 79,643.

^g^Due to the missing data, the total is 81,053.

**Supplementary figure 1.** Survival analysis by Tumor location across decades in the overall cohort. (A) Kaplan-Meier survival curves for RCC and LCC in validation cohort (patients diagnosed between 2004 and 2009); (B) Kaplan-Meier survival curves for RCC and LCC in study cohort (patients diagnosed between 2010 and 2010). 5-yCSS, 5-year cancer-specific survival rate; RCC, right-sided colon cancer; LCC, left-sided colon cancer.

**Supplementary figure 2.** Survival analysis of tumor locations across AJCC stages. The gradient of colors, transitioning from warm to cold tones, represents tumor locations progressing from right to left. Each panel corresponds to a specific stage of the disease. (A) Stage Ⅰ. (B) Stage Ⅱ. (C) Stage Ⅲ. (D) Stage Ⅳ.
