# Supplementary figures and images for: Unraveling the role of tumor sidedness in prognosis of stage II colon cancer
Source: Gastroenterol Rep (Oxf). 2024 Apr 12;12:goae028. doi: 10.1093/gastro/goae028 (PMC11014780; doi:10.1093/gastro/goae028)

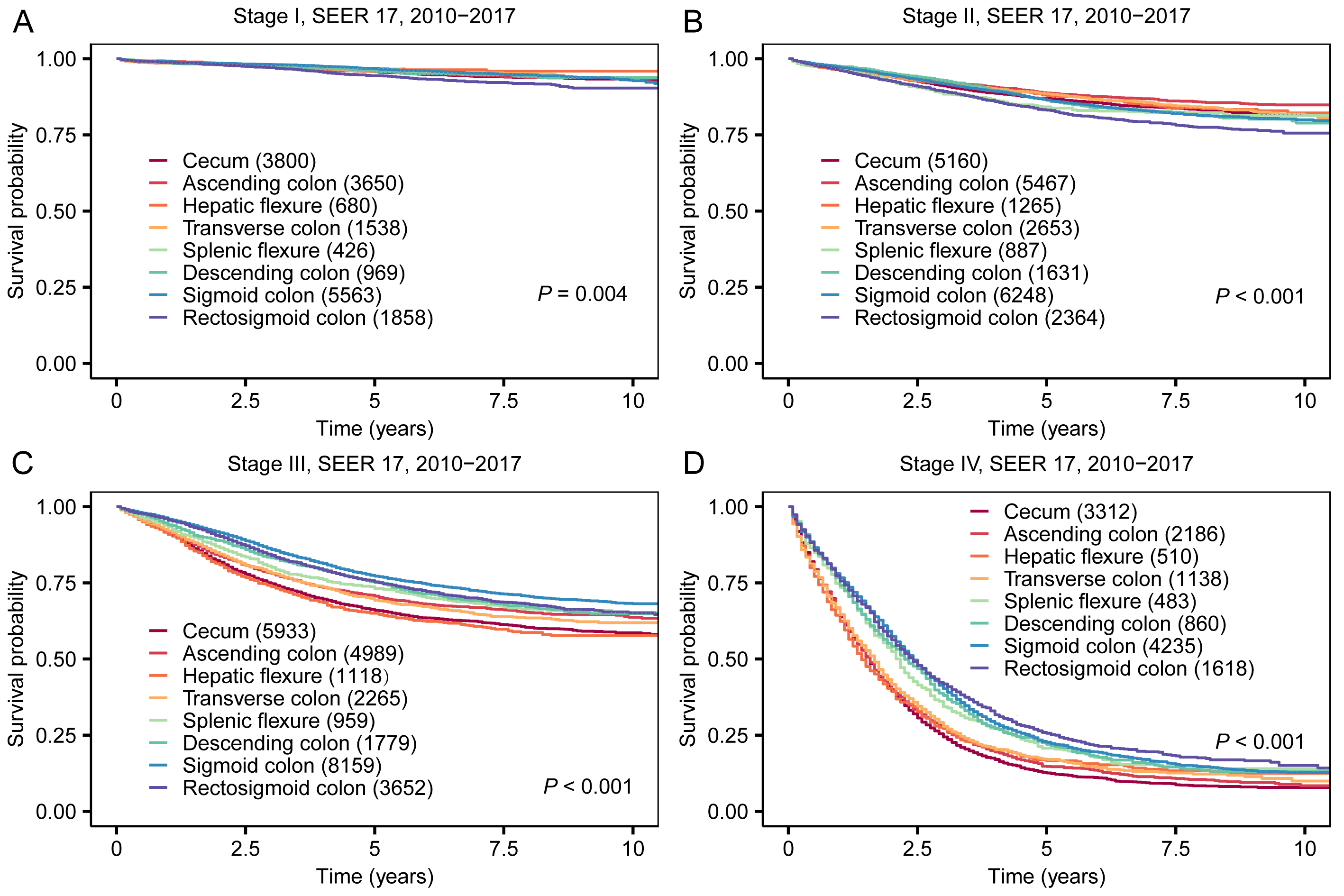

Supplement: goae028_Supplementary_Data [file goae028_supplementary_data.zip › Supplementary Figure 2 final version.tif]
